# Evidence and evidence gaps of medical treatment of non-tumorous diseases of the head and neck

**DOI:** 10.3205/cto000129

**Published:** 2016-12-15

**Authors:** Murat Bas

**Affiliations:** 1 Department of Otolaryngology, Technische Universität München, Germany

**Keywords:** Menière’s disease, specific immune therapy, post-surgery laryngeal edema, ACE inhibitor-induced angioedema

## Abstract

Unfortunately, the treatment of numerous otolaryngological diseases often lacks of evidence base because appropriate studies are missing.

Whereas sufficient high-quality trials exist for the specific immunotherapy of allergic rhinitis and in a limited measure also for the angiotensin-converting enzyme inhibitor induced angioedema, the evidence for Menière’s disease or for pharmacotherapy of postoperative laryngeal edema is rather poor. This contribution will discuss the trial situation and evidence of the respective diseases.

## 1 Postoperative laryngeal edema

Postoperative laryngeal edema may be the result of mechanical pressure caused by intubation pressure or after laryngeal interventions. Besides, also the duration of intubation is an important factor promoting the development of postoperative laryngeal edema. In the literature, postoperative laryngeal edema is also called post-intubation laryngeal edema.

The postoperative laryngeal edema is a feared, possibly life-threatening complication. The risk increases especially after laryngeal surgeries and is well known to ENT specialists. In addition to extended protective intubation and temporary tracheostomy, the application of high-dose cortisone (250–1,000 mg methylprednisolone administered intravenously) is a common prophylactic measure. Now the evidence of this pharmacotherapy is not really clear and shows several gaps.

High-quality original papers about the efficacy of cortisone in this context are very rare. Mostly, those articles deal with retrospective evaluations of partly heterogenic patient populations.

The oldest evaluation dates from 1987 and was performed in France [1]. In the context of a randomized trial, 276 patients were included. Half of them received 40 mg methylprednisolone. In this study, a nasotracheal tube was inserted in all patients. Postoperative laryngeal edema was observed in 6 patients. Four of those 6 patients were in the cortisone group. This means that even more patients developed postoperative laryngeal edema with cortisone than without treatment. With regard to this issue, also several Cochrane analyses were published. In 2000, a Cochrane analysis reported about the prophylactic application of intraoperative cortisone in children and adults [2]. In this context, all randomized and placebo-controlled trials were considered. The main criterion was the question of re-intubation after extubation. Another question was related to the symptom of stridor. Only 7 of 251 trials were qualitatively valuable to be included in the analysis. The patients belonged to different patient populations with regard to their basic disease. Statistically, only the postoperative stridor could be reduced with cortisone prophylaxis in children (n=216: RR=0.53; 95% CI 0.28, 0.97). In 3 trials with adults (n=1,047 patients), the cortisone prophylaxis could neither influence the postoperative stridor nor the re-intubation rate (RR=0.95; 95% CI 0.52, 1.72).

In a Chinese prospective randomized, double-blinded, and placebo-controlled trial with 40 patients, the stridor rate could be reduced after cortisone application, however, it had no impact on the re-intubation rate [3].

Another prospective, randomized, double-blinded, and placebo-controlled study of 64 children describes application of epinephrine and cortisone by inhalation directly before extubation. The result showed that epinephrine and cortisone do not have an impact on the development of laryngeal edema after extubation [4].

In 2009, another Cochrane analysis was performed on the basis of the analysis described above [5]. This time, 11 trials with 2,230 patients were considered as being qualitatively appropriate. Six studies were performed in adults, 2 in newborns, and 3 in children. The population of the newborns was so heterogenic and the application of cortisone was so various that no statistically significant conclusion could be drawn (RR 0.42; 95% CI 0.007 to 2.32). Even the studies with children had very heterogenic populations with partly known airway diseases. With regard to the children with airway abnormalities, the cortisone group showed less stridor (n=62), however, there was no effect in the children with regular airway constellations (n=153). In adults, the prophylactic cortisone application did not have an impact on the re-intubation rate (RR 0.48; 95% CI 0.19 to 1.22). The authors conclude that the prophylactic application of cortisone does not have any positive effect neither in newborns nor in children or adults.

Especially in ENT, there are no trials justifying for example the application of cortisone in laryngeal interventions. In summary, the prophylactic application of cortisone to prevent postoperative laryngeal edema is not evidence-based.

## 2 Prophylactic medication of Menière’s disease

Clinically, Menière’s disease is characterized by recurrent, spontaneous vertigo attacks, fluctuating hearing loss, tinnitus, and pressure in the ear [6]. According to the current knowledge, an endolymphatic labyrinth hydrops causes those symptoms – either because of increased production or disturbed absorption [7]. The high endolymphatic pressure causes recurrent ruptures and leakage of Reissner’s membrane with mixing of low-potassium endolymph with potassium-rich perilymph [8].

The lifetime prevalence of Menière’s disease is around 0.5% [7]. Most commonly, the disease starts on one side, the frequency of attacks varies importantly. In the further course, 50% of the patients develop bilateral Menière’s disease [9]. This fact also explains why Menière’s disease is considered as the second most frequent origin of bilateral vestibulopathy. 

Since the origins of endolymphatic hydrops – and thus Menière’s disease – are mostly unknown, targeted causal treatment is not possible. Pharmacotherapy aims at treating the vertigo attacks in the acute stage and furthermore to avoid attacks of Menière’s disease with vertigo and hearing loss [7].

The current therapy of Menière’s disease is based on two principles:

### 2.1 Treatment of acute attacks

Vertigo and nausea can be reduced by application of antivertigo agents as they are also administered for the treatment of other acute disorders of the labyrinth function, e.g. dimenhydrinate 100 mg as suppository [7].

### 2.2 Prophylactic treatment

The aim of prophylactic treatment is to reduce the endolymphatic hydrops. Despite the high prevalence of Menière’s disease and many clinical trials, there is no therapy up to now that is proven to be effective. The spectrum of recommendations reaches from salt-free nutrition, diuretic agents, transtympanic application of gentamicin (20–40 mg in intervals of several weeks until the symptoms improve) or betahistine up to surgical procedures [7]. Positive effects on the incidence of attacks were published for the transtympanic instillation of gentamicin [9] and high-dose, long-term application of betahistine dihydrochloride (3×48 mg per day for 12 months). The direct origin of Menière’s disease, i.e. the endolymphatic hydrops is often treated with betahistine. However, already in 2001 a systematic review (meta-analysis) of the Cochrane Collaboration revealed that there are not sufficient data to judge if betahistine has a real effect in the context of Menière’s disease [10], [11]. The skepticism regarding the application of betahistine has become more and more important since it is meanwhile possible to quantitatively measure the extension of the inner ear vessels filled with endolymph by means of magnet resonance imaging (MRI). In an according evaluation of 6 patients, betahistine showed no effect in none of the patients [10]; and a case study of a female patient over 2 years revealed the end of vertigo attacks but a deterioration of the hydrops and hearing capacity in both ears [10].

More recent studies postulate a clinical improvement of the symptoms of Menière attacks after application of high-dose, long-term betahistine therapy [12], [13], [14], [15]. A therapy recommendation is based on observations of 112 patients who received the medication either 3×16 mg/d or 3×24 mg/d or 3×48 mg/d for at least 12 months [15]. The higher dosage led to a significant reduction of the incidence of the attacks and was well tolerated. However, this trial was not randomized and not blinded. The results that were observed in this context have to be confirmed in a randomized and blinded study.

In cases of severe and frequent attacks (more than 2 in 3 months), treatment with a low-dose loop diuretic (e.g. Furosemide) can be attempted [16]. Also in this context it must be emphasized that the effect has not been proven up to now [16]. Only two clinical studies have yet been published, while only one of those studies was double-blinded and placebo-controlled. In this study, the diuretic therapy led to a reduction of the subjective vertigo attacks, but it had no effect on hearing loss and tinnitus [17], [18].

Circulation-enhancing by medication or compressed-air chambers are often applied for inner ear complaints such as sudden hearing loss or tinnitus, however, even in cases of confirmed diagnosis of Menière’s disease, those measures have no effect. Even other highly promoted procedures such as low-level laser therapy, where the auricle is irradiated with red light laser, must be questioned because laser light – that is said to have a positive effect on the sensory cells of the inner ear – physically does not reach this area.

During the last years, intratympanic injection of gentamicin and/or cortisone has been applied again and again in trials in different combinations. The objective of such treatments was to eliminate the vertigo attacks and to preserve the hearing capacity. According to a study performed in 2011, injection of gentamicin into the middle ear seems to reduce the vertigo attacks, however, the hearing loss cannot be avoided. Injection of cortisone has no impact on the occurrence of vertigo attacks [19]. 

## 3 Conclusion

Prophylactic pharmacotherapy of Menière’s disease is not sufficiently verified. The effect of certain drugs was not proven, it is only possible to reveal certain effect tendencies on isolated symptoms. The long-term treatment with betahistine shows a positive effect on vertigo-attacks (evidence level 3), however, there is no impact on hearing loss and tinnitus.

## 4 Angioedema induced by angiotensin converting enzyme

### 4.1 Etiology and pathophysiology of angioedema

**Figure 1:**
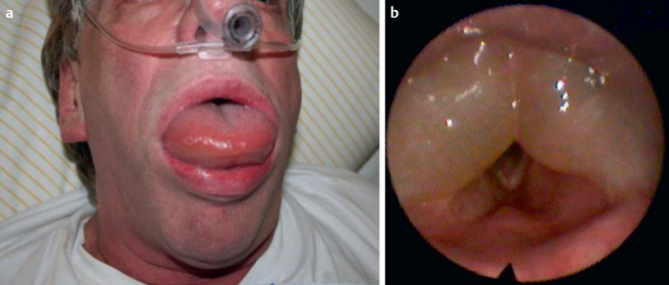
ACE inhibitor-induced angioedema of the tongue and the larynx

Angioedemas are edematous swellings of deeper tissue layers affecting the skin as well as the mucosa (Figure 1 (Fig. 1)).

**Figure 2:**
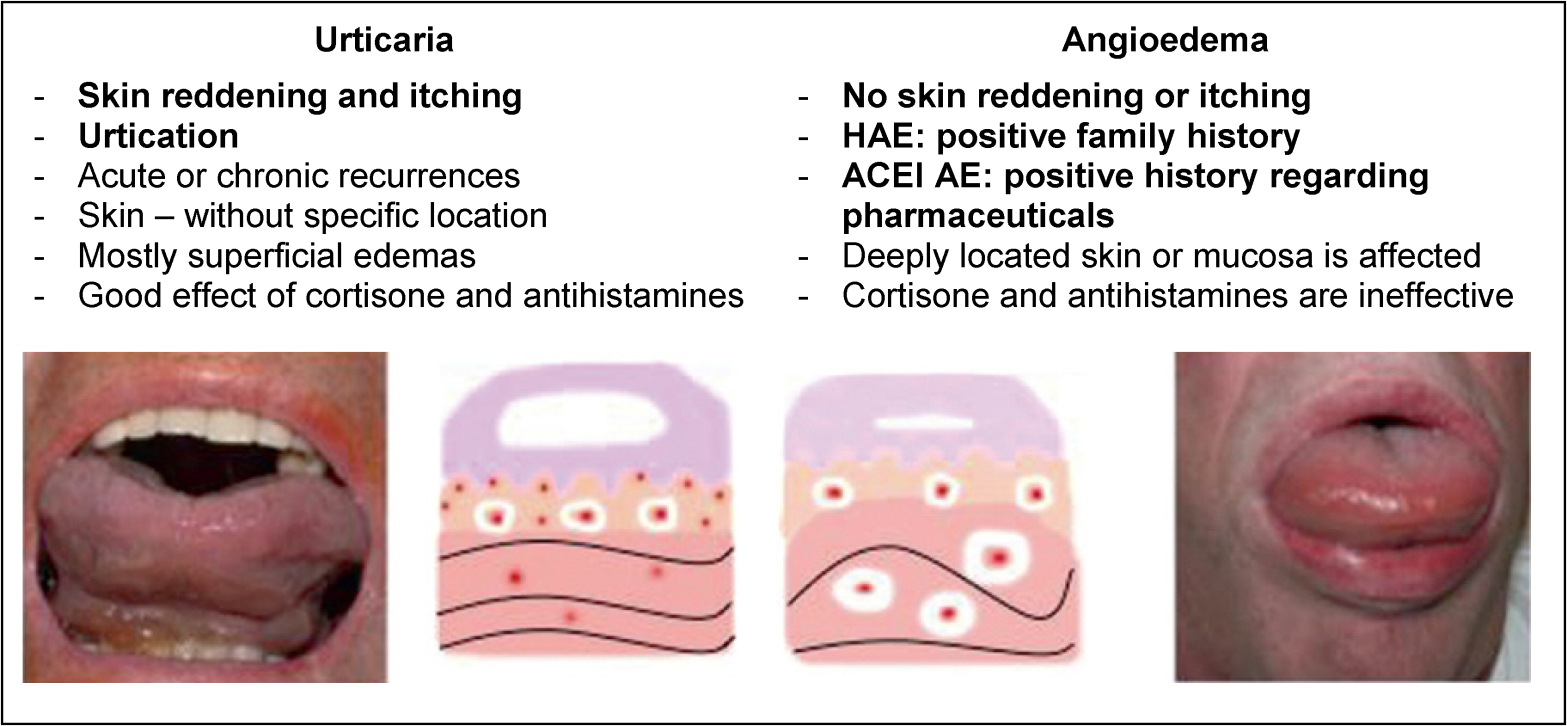
Characteristics of angioedema in comparison to urticaria

Manifestations in the area of the airways may impair breathing and in rare cases they may even lead to suffocation [20]. Generally, angioedemas are classified into allergic (urticaria) and non-allergic angioedemas [21]. In contrast to allergic urticaria, it is typical for the non-allergic manifestation that skin reddening and in particular itching are missing. While histamine is the pathophysiological mediator of allergic urticaria, non-allergic angioedemas are mainly triggered by the effect of bradykinin as mediator (Figure 2 (Fig. 2)).

This difference is crucial for a rationally sound therapy of the disease. One particular problem is chronic urticaria. Angioedemas that may develop in the course of the disease have an allergic genesis in only 10% of the cases [22], [23]. Hence, such angioedemas only rarely respond to anti-allergic standard therapies such as antihistamines and corticosteroids. Because of missing reddening of the skin or itching, it is often difficult to define the difference between angioedemas based on chronic urticaria and non-allergic angioedemas. Those difficulties in finding the correct diagnosis frequently result in an inadequate treatment of the patients. Bradykinin-induced angioedemas either result from increased bradykinin production or from inhibited bradykinin metabolism. Bradykinin is a nonapeptide that is physiologically produced within the kallikrein-kinin system. The history of the detection of the kallikrein-kinin system started more than 100 years ago when Abelous and Bardier discovered the blood pressure lowering effect of urine [24]. Kinins are pharmacologically active peptides that are released by kallikrein from kininogens into body fluids and tissue. In contrast to this, the C1 inhibitor (C1-INH) works as endogenous kallikrein inhibitor and limits the synthesis of kinin. In addition to bradykinin, also kallidin and methionyl-lysyl-bradykinin belong to the family of kinins. The last mentioned are transformed by amino-peptidases contained in the plasma and urine to bradykinin [25].

The kallikrein-kinin system is associated with the renin-angiotensin-aldosterone system (RAAS) and partly antagonizes its effects. The functional linking between both systems is based on the non-specificity of the angiotensin-converting enzyme (ACE) that on the one hand produces angiotensin II and on the other hand degrades kinins such as bradykinin or substance P to inactive metabolites [26], [27]. Beside ACE, other proteases (aminopeptidase P, dipeptidylpeptidase IV, carboxypeptidase N) contribute to the metabolism of bradykinin. If those enzymes are inhibited in addition to ACE, further increase of the bradykinin concentration in the plasma and tissue must be expected [28], [29], [30].

### 4.2 Bradykinin receptors

Bradykinin receptors are G protein-coupled receptors ubiquitously located on the cell surface. Up to now, 2 receptor subtypes could be identified, i.e. the bradykinin receptor type 1 (BKR-1) and 2 (BKR-2). They have different pharmacological properties [31], [32], [33], [34], [35], [36], [37]. The human gene for BKR-2 was located on the chromosome 14q32 [38] whereas BKR-1 is found on chromosome 14q32.1-q32.2 [39]. The amino acid sequences of BKR-1 and BKR-2 only have a homology of 36% [37]. BKR-1 is synthesized de novo by many organs as reaction to tissue damage while BKR-2 is generally expressed constitutively [39], [40], [41].

### 4.3 Effects of bradykinin

In the 1980ies, the detection of different selective antagonists of BKR-1 and BKR-2 allowed important functional examinations of the role of kinins [34].

Recently, the development of C1-INH and BKR-2 transgenic mice led to important knowledge about the role of kinins in vivo [42], [43]. So for example the effect of bradykinin on the vascular permeability could be revealed by targeted inhibition of C1-INH [43]. Already earlier it was possible to show that bradykinin dilates peripheral and coronary arteries, that it may reduce arterial blood pressure in normotensive animals, and that it has anti-thrombogenic, anti-proliferative, and anti-fibrinogenic properties [41], [44], [45], [46], [47], [48].

According to the current knowledge, the cardio-vascular effect of bradykinin is mediated by activation of BKR-2 on endothelial cells which leads to a release of nitrogen monoxide (NO), prostaglandin PGl2, and the tissue-type plasminogen activator [49], [50]. It could also be shown that bradykinin is involved in the cardio-protective effect of “preconditioning” in myocardial ischemia or reperfusion injury [51]. Bradykinin can reduce the extent of infarction [52], [53] and limit the growth of cardiomyocytes [54], [55]. Furthermore, kinins can constrict the smooth bronchial muscles [56] which allows the conclusion that dry cough induced by ACE inhibitors is mediated by bradykinin and also substance P [57], [58]. Furthermore, the local accumulation of bradykinin may activate pro-inflammatory peptides and release local histamine possibly leading to hypersensitivity of the cough reflex [59]. Finally, it could be proven that bradykinin increases the release of insulin from the pancreatic B cell. This effect is mediated by the increase of intracellular calcium response on hyperglycemia [60], [61]. Bradykinin also increases the insulin-depending transportation of glucose [62]. Further examinations revealed that locally released bradykinin increases the uptake and availability of glucose in target tissue independently from insulin secretion [63], [64]. Based on those findings it is most probable that the reduced metabolism of bradykinin contributes to the positive properties of the ACE inhibitor in patients suffering from cardiovascular diseases. This includes for example the reduction of diabetes-related consequential damage or the reduction of new cases of type-2 diabetes mellitus [65].

### 4.4 Bradykinin-induced angioedema

#### 4.4.1 Hereditary angioedema

In 1882, Heinrich Irenäus Quincke described an acute and clearly defined edema. Even if such an edema was already known from earlier case descriptions, it was Quincke who accurately described this disease and differentiated it from urticaria [66]. Today, so-called Quincke’s edema is a synonym of angioedema and is used as generic term for description of defined edema without urticaria and/or pruritus. 

According to the current knowledge, the lack of serine protease C1-esterase inhibitor (C1-INH) that is due to a genetic effect plays a causal role in the development of HAE [67]. This leads to a series of alterations within the complementary system, which has also diagnostic significance. It is crucial that an important physiological inhibitor for the production of bradykinin is missing because of a lack of C1-INH because C1-INH is an endogenic inhibitor of kallikrein. The clear reduction of the C1-esterase inhibitor (C1-INH) activity leads to an increased production of bradykinin. The human C1-INH gene was found on chromosome 11 (11a12-q13.1) [48]. Two different variants of HAE have been described: HAE type 1 with reduced C1-INH level and a deficient function (85% of all cases) and HAE type 2 with regular protein concentration but functional deficit (15% of all cases). It is the case of heterozygous autosomal dominant inheritance with an incidence of 1:50,000, independent from ethnicity or sex [68] In 2006, another origin of autosomal dominant inheritance of HAE was described, which are mutations in exon 9 of the F12 gene that lead to amino acid position 309 of the coagulation factor XII for substitution of threonine by lysine or arginine [69]. The result of those activating mutations is – comparable to the classic HAE type 1 and 2 – an increased kinin production based on an increased enzymatic activity of the coagulation factor XII. Activated factor XIIa transforms pre-kallikrein into kallikrein, which accelerates the transformation of high molecular weight kininogen (HMWK) to bradykinin. In a family mostly women are affected; the intake of estrogen is a significantly precipitating factor. This circumstance may be explained by the fact that the coagulation factor XII is synthesized depending on estrogen. Accordingly, women who take estrogen-containing medication as for example the pill or who are pregnant are especially at risk because they have additionally increased factor XII serum concentrations beside the activating mutation. Very rarely, also male patients could be identified, however, their attacks occur less frequently and with lower intensity. The most frequent symptoms of the patients suffering from HAE type 3 are swellings of the face (93%) and the tongue (54%) as well as abdominal pain attacks (50%). Laryngeal (25%) and uvula edema (21%) are often observed [70]. In contrast to type 1 or 2, the quantitative and functional values of C1 esterase inhibitor are regular. However, mutations in exon 9 of the F12 gene can only be identified in part of the patients. So it can be expected that there are other genetic reasons for this subtype that are currently still unknown. Recently the first case of homozygous C1-INH lack with a mutation of c.1576 T>G was reported [69]. In mice, the targeted interruption of the C1-INH effect led to an increased vascular permeability that could be reversed by the treatment with human plasma pool C1-INH [43]. Other findings that were revealed in this transgenic murine population brought further convincing proofs for a significant involvement of BKR-2 in the pathogenesis of angioedema. So for example the increased vascular permeability could be reduced drastically by treatment with the BKR-2 antagonist icatibant (see below).

#### 4.4.2 Triggering factors

Patients with HAE report about a multitude of factors that trigger angioedema attacks. Those are among others exposure to cold, mechanical trauma (e.g. tissue compression, sitting or standing for a longer time), certain food products (e.g. eggs, alcohol), infections, concomitant diseases, contact with pesticides or other chemicals, excitement, stress, and certain drugs such as ACE inhibitors and estrogens [67]. Those rather anecdotic reports, however, have never been evaluated systematically and seem to be influenced by individual patient characteristics. One exception in this context is the intake of estrogen-containing contraceptives and estrogen products for hormone replacement therapy. It could be shown that some female HAE patients react with an increased incidence of attacks when the serum estrogen is increased during the menstruation cycle, during pregnancy or because of contraceptives or postmenopausal hormone replacement therapy [70], [71]. Other researchers found similar cases in male and female patients who underwent antiandrogen therapy with cyproterone [72]. Even pharmaceutics inhibiting the metabolism of bradykinin may cause an increased incidence of attacks – hence ACE inhibitors are contraindicated in HAE patients [73], [74].

#### 4.4.3 Iatrogenic bradykinin-induced angioedema

**Figure 3:**
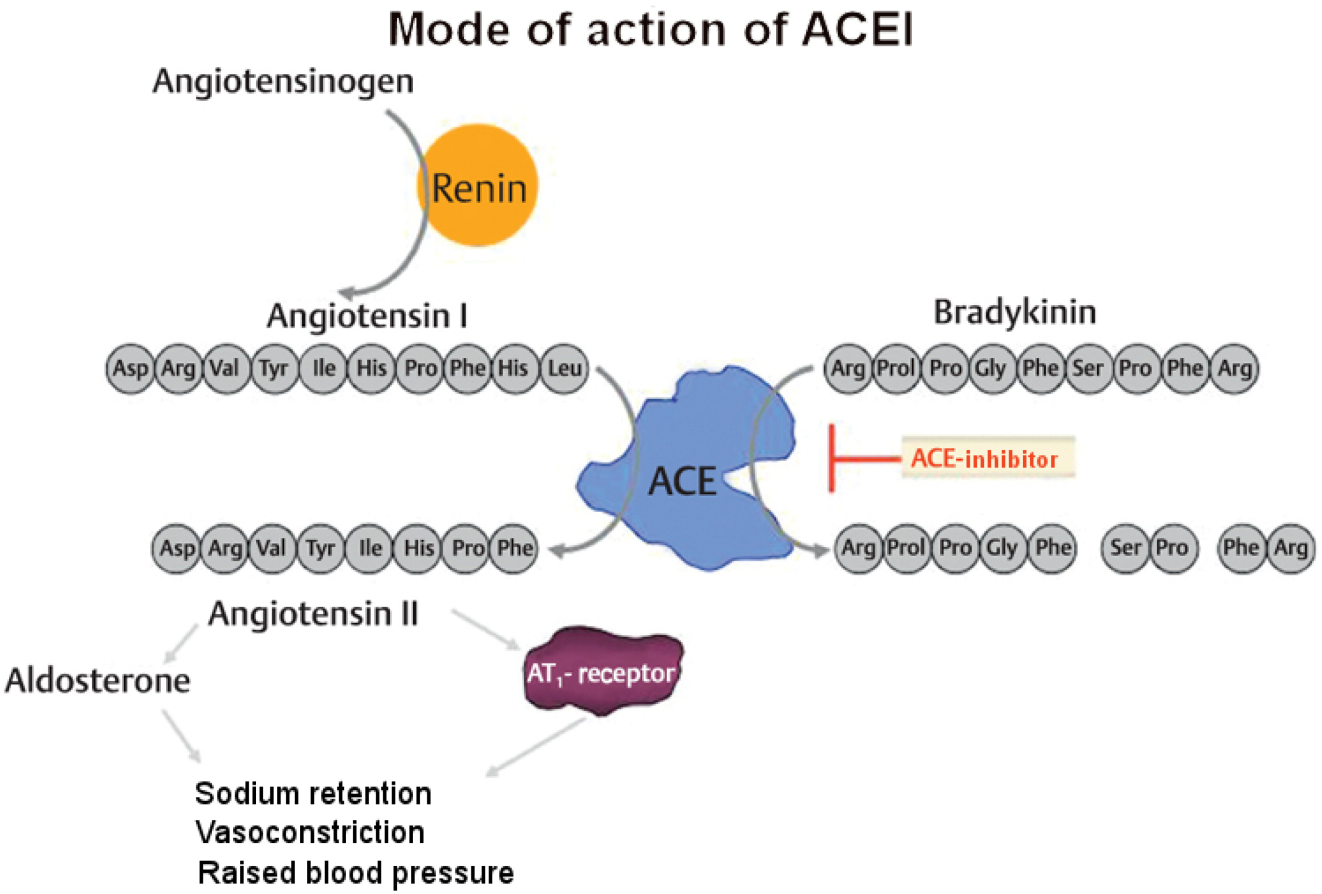
Mode of action of the ACE inhibitor

The most frequent reason for the occurrence of bradykinin-induced angioedema is the intake of pharmaceutics that lead to an inhibited metabolism of bradykinin. Those are not only ACE inhibitors such as enalapril but also AT1 blockers as losartan and the renin inhibitor aliskiren. The reduced metabolism of bradykinin induced by this group of drugs is the desired effect for therapy of cardiovascular diseases. However, it can be assumed that ACE inhibitors have a stronger inhibiting effect on bradykinin metabolism than other substance groups (Figure 3 (Fig. 3)).

#### 4.4.4 ACE inhibitor-induced angioedema

One characteristic of ACE inhibitor-induced angioedema is the regular manifestation in the area of the airways. Swellings in the head and neck region, especially pharynx and larynx, often require an inpatient treatment for several days and sometimes even intensive care is needed. 

The incidence of ACE inhibitor-induced angioedema varies according to the different examinations, probably because of ethnical differences. So the incidence of ACE inhibitor-induced angioedema in Caucasians amounted to 0.1–0.7% [20], [67], [68], [75], [76], whereas the susceptibility of colored Americans was much higher [77]. The current meta-analysis of the side effects of pharmaceutics for treatment of cardiovascular diseases in those patients revealed a relative risk for the manifestation of ACE inhibitor-induced angioedema that was three times higher than in white Americans [24]. Among the nearly 7 million patients treated with ACE inhibitors in Germany and an assumed rate of angioedema of 0.3–0.5%, around 20,000-35,000 cases have to be expected annually. This would mean a calculated incidence of 1:4,000, which means that ACE inhibitor-induced angioedema occurs much more frequently than HAE [78]. If other enzymes are inhibited beside ACE catalyzing the metabolism of bradykinin, an even higher bradykinin concentration must be expected [28], [29], [30]. This effect is obvious in the application of omapatrilat that inhibits neutral endopeptidase in addition to ACE. During the clinical testing phase, the comparison to the ACE inhibitor enalapril revealed a more than three times more frequent occurrence of angioedema (2.17 vs. 0.68%) so that finally this agent was not approved [59].

#### 4.4.5 AT1 blocker-induced angioedema

Angioedema is more rarely caused by AT1 blocker than by ACE inhibitors [76], [78]. In the VALIANT trial, about 4,900 patients for each study arm were treated over 2 years with valsartan, captopril, or valsartan and captopril. While 0.2% of the patients of the valsartan group developed angioedema, 0.5% of the patients of each of the other two groups showed this side effect. However, in view of the mechanism of action it first seems to be surprising that also this angioedema is induced by bradykinin. Also the fact that the combined application of valsartan and captopril did not cause more angioedema than captopril alone might contradict to an involvement. But in the context of a recent study it could be shown that AT1 blockers increase the bradykinin level in hypertensive patients [79], [80]. The blood levels of angiotensin II increased under the treatment with AT1 blockers because those agents interrupt the physiological feedback mechanism that regulates the synthesis of angiotensin II via the release of renin [81]. At the same time, all AT type 1 receptors are blocked allowing the activation of more AT type 2 receptors by angiotensin II. In this context it seems to be important that the stimulation of AT type 2 receptors leads to an inhibition of the ACE activity via a still unknown mechanism [82]. So the increased bradykinin concentration in plasma observed by Campbell et al. after the application of AT1 blockers might be based on an inhibition of the bradykinin metabolism [80]. Based on earlier animal experiments, further evaluations are necessary to confirm the mentioned hypothesis [83]. Nonetheless, AT1 blockers should not be applied in patients who have already had an ACE inhibitor induced angioedema [84]. 

#### 4.4.6 Renin-inhibitor-induced angioedema

Another pharmacological alternative to influence the renin-angiotensin-aldosterone synthesis is the application of the relatively new renin inhibitor aliskiren that is also admitted for the treatment of hypertonia. The inhibition of renin neither leads to a direct inhibition of ACE nor to an increased activation of AT type 2 receptors and thus probably not to an increased bradykinin concentration in the plasma. However, in the approval study, aliskiren did not turn out to be superior to ACEI or sartans [85], [86], [87], [88]. In the approval studies, patients suffering from mild or moderate hypertonia were included. In contrast to other antihypertensive agents, a reduction of the cardiovascular morbidity or mortality could not be proven for aliskiren [85], [86], [87], [88]. For patients having had angioedema induced by ACE inhibitors or sartans, aliskiren does not represent an alternative because this circumstance was classified as contraindication for therapy by the official authorities.

#### 4.4.7 Acquired angioedema

Acquired angioedema (AAE) develop on the basis of non-genetic lack of C1-INH and mostly concern adults [87]. It can be induced for example by a severe basic disease such as malignant lymphoma. Patients with lympho-proliferative diseases may develop angioedema that correlates with a reduced C1-INH plasma concentration and activity [89], [90]. In contrast to HAE with deficient C1-INH synthesis and/or activity, the AAE is characterized by the fact that a high number of idiotype-anti-idiotype immune complexes (autoantibodies) is present that consume C1q molecules and afterwards C1-INH [91]. Other diseases such as hepatocellular carcinoma and liver cirrhosis can be associated with reduced C1-INH plasma concentration, but in those cases angioedema has never been described. On the other hand, one case of a lymphoma-associated angioedema is known where the C1-INH plasma concentration was regular [92]. Recently a new C1-INH mutation was described that was associated with a significantly inhibited C1-INH secretion of the monocytes [92]. 

## 5 Therapy of acute ACE inhibitor-induced angioedema

The particular risk of angioedema of the head and neck is the obstruction of the airways leading to suffocation. With this regard it is necessary to clarify during each contact with the patient where exactly the angioedema is located, how severe the disease is, and especially if the airways are acutely at risk requiring immediate intervention. Generally, the following concepts are available for therapy of angioedema of the head and neck:

- Mechanical securing of the airways
- Supportive measures of therapy
- Symptomatic pharmacotherapy
- Causal pharmacotherapy

### 5.1 Mechanical securing of the airways

Mechanical securing of the airways must be considered when respiratory insufficiency is diagnosed:

- Inability to swallow (saliva runs out of the mouth)
- Inspiratory stridor
- Cyanosis

If one of these symptoms is observed, securing of the airways has to be immediately performed.

#### 5.1.1 Types of mechanical securing

**Figure 4:**
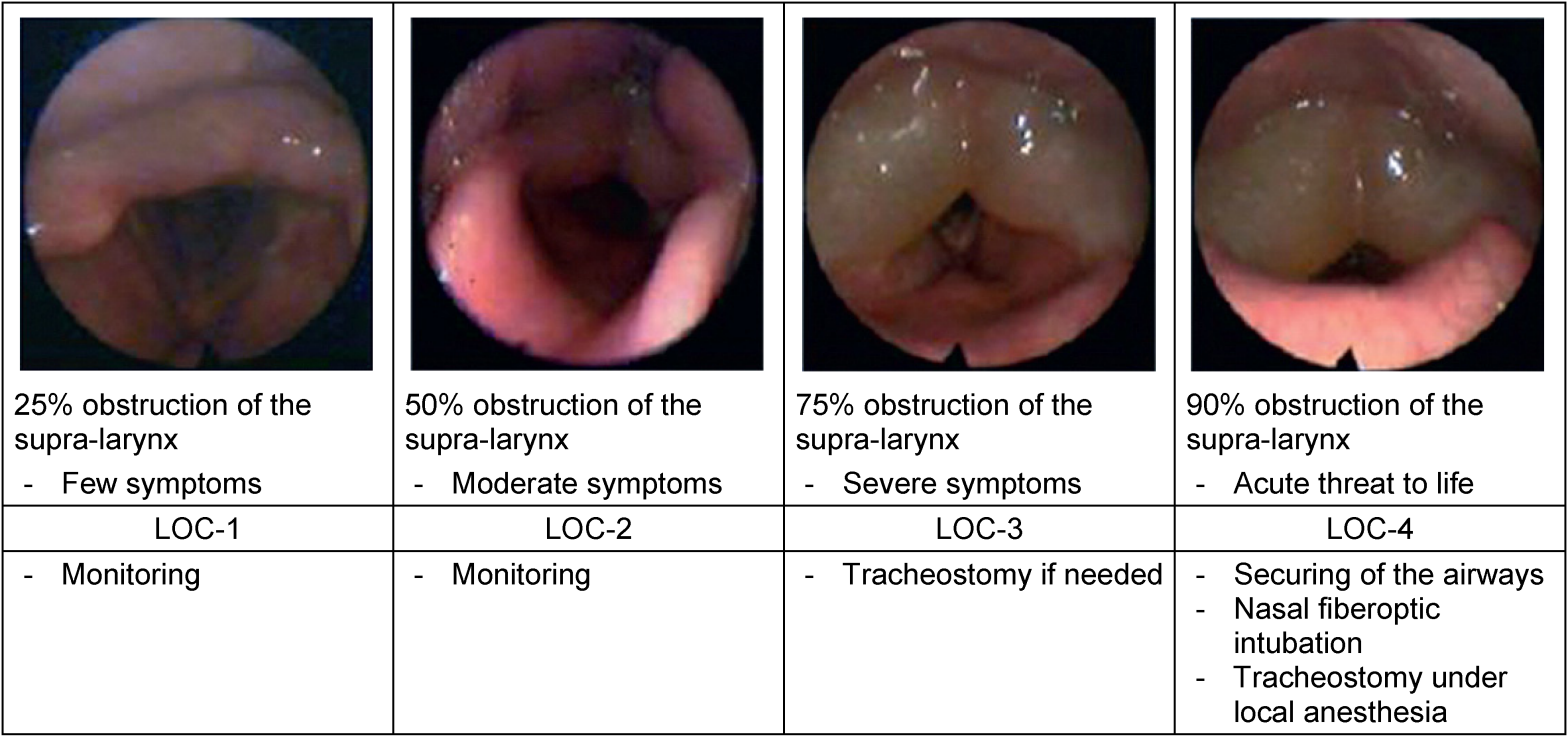
Classification of laryngeal edema

**Figure 5:**
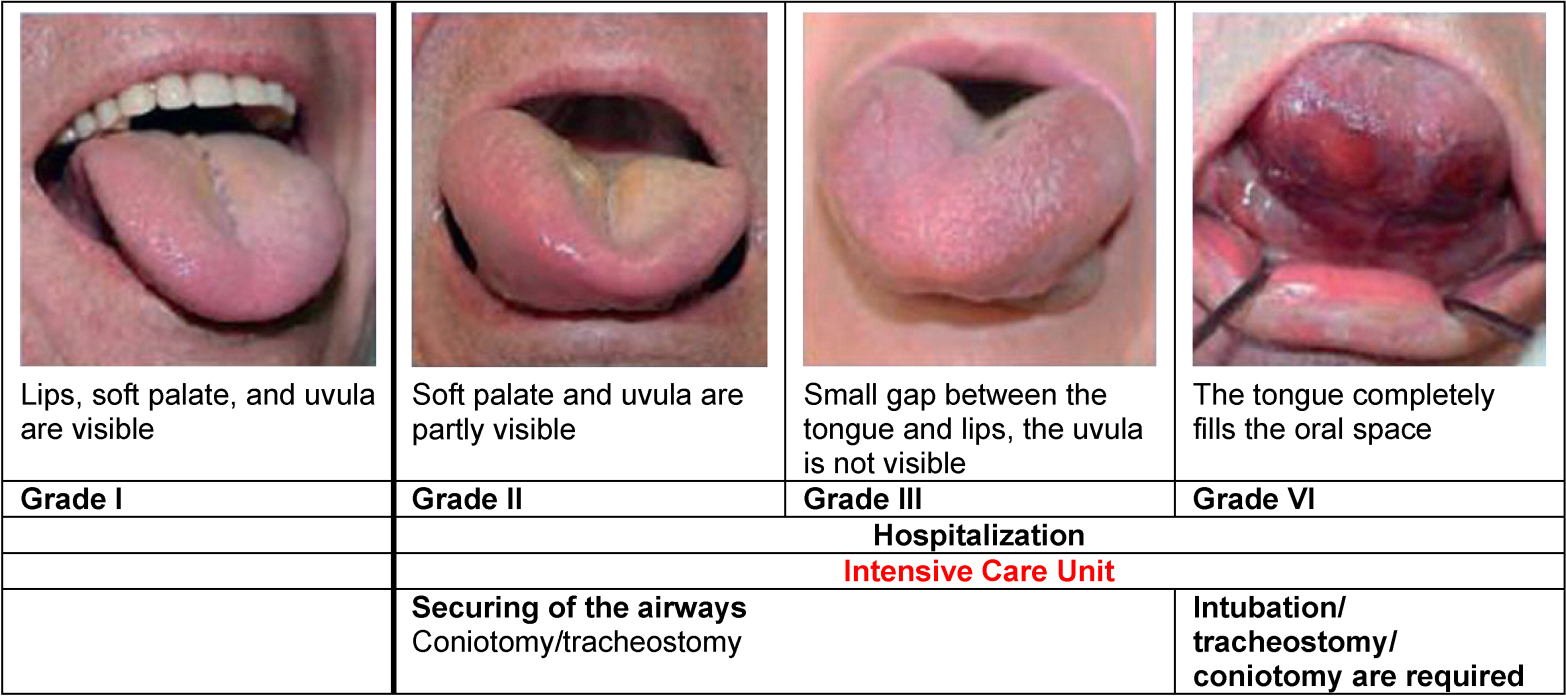
Emergency management in the case of lingual angioedema

Depending on the location of the angioedema, the decision for a specific method must be taken (Table 1 (Tab. 1)). Beside the above-mentioned symptoms, the degree of severity/the size of the angioedema is important for the technique. The classification of the laryngeal (Figure 4 (Fig. 4)) and lingual angioedema (Figure 5 (Fig. 5)) can be helpful to find the right decision. Because of the considerable tissue damage in the area of the upper airway and swallowing tract, the mechanical intervention may lead to a prolonged angioedema attack, sometimes even lasting for several days.

#### 5.1.2 Supportive measures of therapy

In the context of angioedema in the area of the tongue and the oropharynx, sucking ice cubes may be helpful (vasoconstriction, decongestive effect). In cases of circulation problems, the application of infusions (sodium chloride/Ringer) is possible. The upright position of the upper part of the body is also useful.

#### 5.1.3 Symptomatic pharmacotherapy

All agents that are effective for the therapy of angioedema attacks can be applied. The administration of other vasoconstricting agents such as epinephrine as inhalation/spray can also be applied (off-label use; dosage up to 8 mg per application). However, there are no trials regarding the efficacy. Thus, the supportive treatment of bradykinin induced angioedema with epinephrine is not evidence-based. In cases of pains, the application of analgesics as additional medication is possible.

#### 5.1.4 Pharmacotherapy without efficacy proof

In cases of ACEI induced angioedema, no officially approved pharmacotherapy is available. Despite the missing approval (off-label use) ACEI induced angioedema were treated with cortisone (250–500 mg methylprednisolone) and antihistamines (clemastine 2 mg) in the past. There are neither studies nor case series supporting the efficacy of this treatment scheme. In one case series [93] the clinical course with cortisone and the time of complete healing of the edema were analyzed retrospectively. Under cortisone therapy, 3/47 patients had to undergo tracheostomy because of missing improvement, 2/47 were intubated, and 12/47 received a second cortisone application. The time of complete healing of the edema was 33 hours and was equal to placebo administration. A recently published double-blinded trial revealed an average of 27 hours until complete healing of the edema with cortisone and antihistamines and thus confirmed the missing effect of cortisone and antihistamines in the treatment of angioedema.

*Conclusion:* The application of cortisone and antihistamines in the context of angioedema is not evidence-based. Trials could not confirm a reliable effect [94].

#### 5.1.5 Causal pharmacotherapy

Beside agents for circulation and respiration emergencies, specific causally effective drugs are available. Those are pharmaceutics that concern the pathophysiology and either avoid, stop, or attenuate the disease. In the context of bradykinin-induced angioedema, they either inhibit the bradykinin production or they impede its effect on its receptor.

The non-allergic, bradykinin-induced angioedema generally does not respond to antihistamines and corticoids in contrast to the allergic angioedema. A specific therapy of bradykinin-induced angioedema aims at avoiding the progression of the swelling to other levels of the head and neck area and to reduce the already existing symptoms as rapidly as possible.

### 5.2 Icatibant

**Figure 6:**
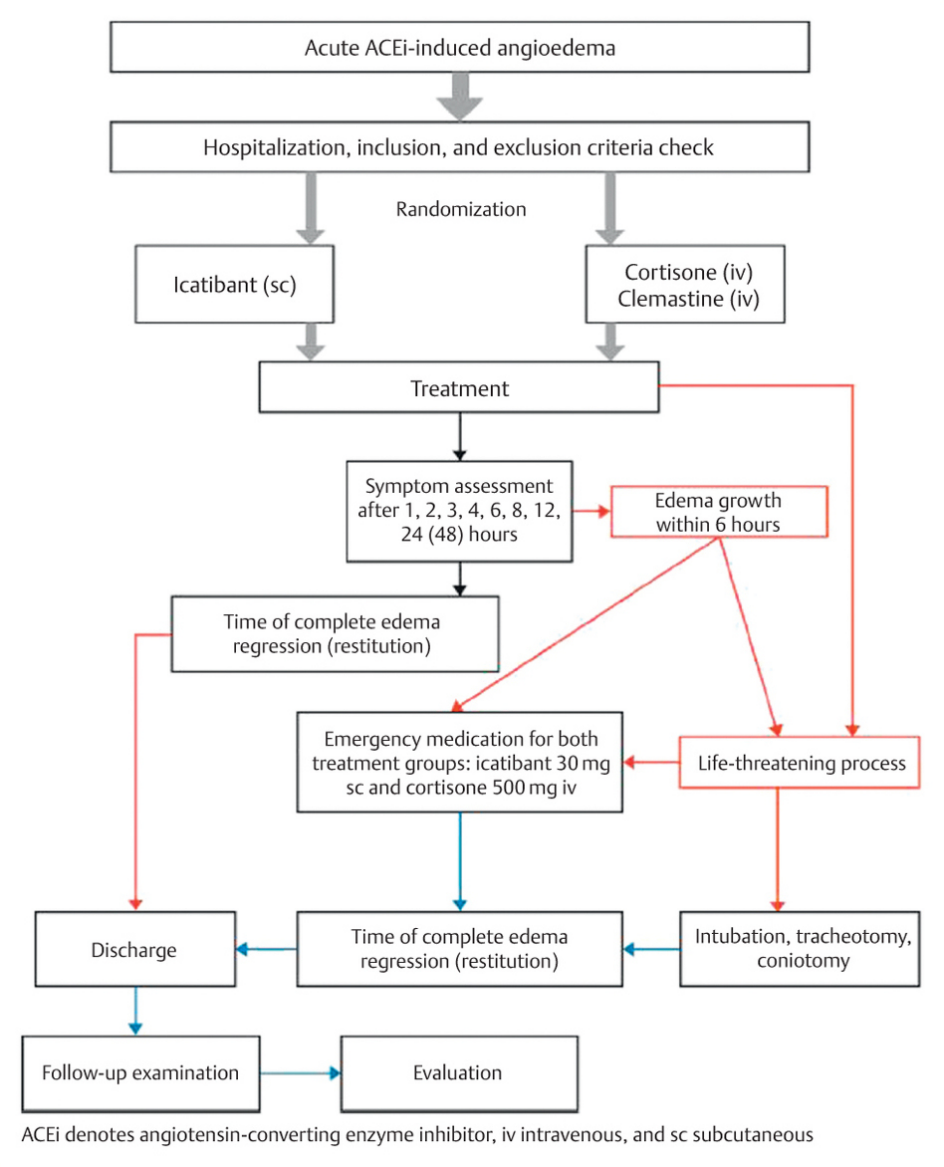
Study design of AMACE

Since the majority of non-allergic angioedema is based on a pathological increase of the tissue hormone bradykinin, a reliable efficacy of the synthetic bradykinin B2 receptor antagonist icatibant (Firazyr™) can be expected. Icatibant acts as a bradykinin inhibitor by blocking the binding of native bradykinin to the bradykinin B2 receptor. Currently, icatibant is approved for symptomatic treatment of HAE angioedema in adults in the European Union. Icatibant (30 mg) is administered subcutaneously in the area of the abdomen, a first palliation of the symptoms is expected already after a median interval of about 45 minutes. Up to now, no systemic side effects have been observed, there is only the description of a transitory erythema at the injection site. Since its approval, meanwhile dozens of patients suffering from ACE inhibitor-induced angioedema were treated successfully off-label. A total of 4 original papers were published on this topic. Three of those publications are case series encompassing 33 patients. The patients in these case series had received partly cortisone or they had been intubated. In all 3 case series, the successful treatment with icatibant was confirmed. The complete healing of the angioedema was achieved after an average of 4–5 hours. It took more than 33 hours to completely heal the angioedema with formerly applied therapy of cortisone and antihistamines [93]. The 4^th^ original paper of our group is a double-blinded, two-arm, and randomized trial that was conducted as multicenter study. A total of 32 patients were screened and 30 of them were randomized afterwards. The patients received either icatibant and placebo or the standard therapy (cortisone and antihistamines) and placebo (Figure 6 (Fig. 6)).

Half of the patients underwent acute therapy with 30 mg icatibant subcutaneously injected into the abdominal wall, the others were treated with the off-label standard therapy of 500 mg prednisolone (intravenous application) (Solu Decortin H, Merck) with 2 mg clemastine (Tavegil, Norvartis).

In order to analyze the pharmaceutics in an overall assessment, 3 rankings were performed: The patients assessed the intensity of 6 symptoms (pain, dyspnea, dysphagia, voice changes, foreign body sensation, and sense of pressure) on a visual analogue scale (VAS) from 0 (not present) to 10 (maximum intensity). This questionnaire was filled out before therapy and in several time intervals after application of the therapeutic medication. The examiner assessed the severity of the mentioned 6 symptoms based on a specific evaluation scale. Furthermore, the examiner described the severity of the angioedema at four different locations: lips/cheek, tongue, oropharynx, and hypopharynx/larynx ranking from 0 (no angioedema) to 4 (severe swelling). The primary endpoint was the time of complete healing from the angioedema. 

Based on those analysis, the interval starting at the time of injection of the study medication up to the complete regression of the symptoms was assessed that represented the primary endpoint of the trial. Also the onset of symptom relief was an important criterion. Additionally, a comparison was made of the numbers of patients of both groups that did not respond to the therapy. In such a case, the patients received an emergency treatment consisting of 30 mg icatibant with 500 mg prednisolone, regardless the group to which they belonged. The evaluation of this study shows that icatibant is clearly superior to current standard therapy with prednisolone and clemastine. Finally, all patients of both cohorts had a complete resolution of the edemas, but 3 patients of the standard therapy group had to undergo the mentioned emergency therapy because of primary therapy resistance, which never happened after icatibant therapy. Due to a complicated course, tracheostomy had to be performed in one patient of the prednisolone/clemastine group.

**Figure 7:**
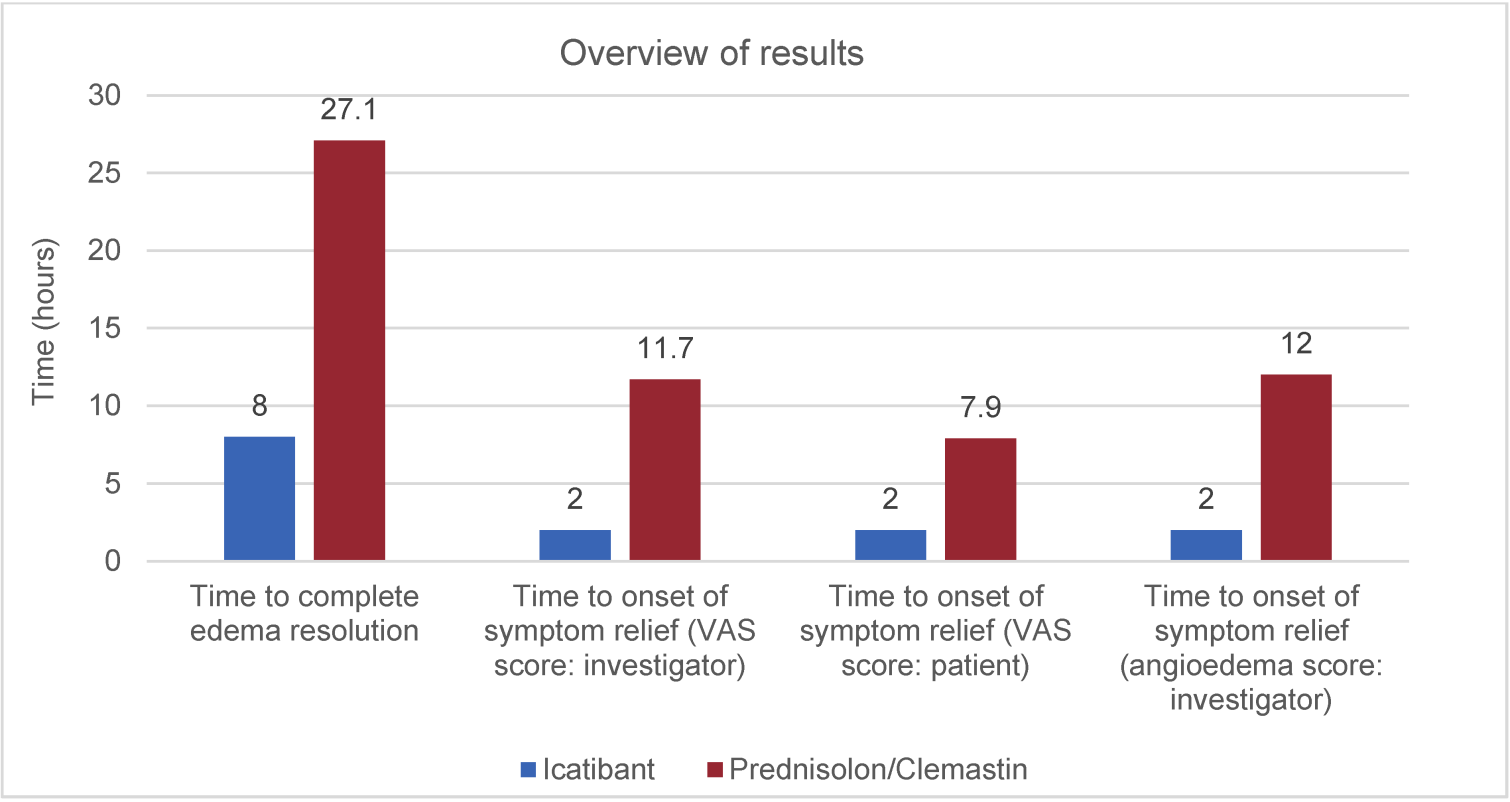
Overview of the results of the AMACE study comparing icatibant (blue) and prednisolone/clemastine (red)

The median time of the icatibant cohort to complete resolution of the angioedema was 8 hours, which was 70% shorter than the control group with standard therapy – they needed 27.1 hours (p<0.002). Five patients of the icatibant group observed complete regression within 4 hours, which did not happen once in the prednisolone/clemastine group. Furthermore, the onset of symptom relief was significantly earlier: on the average the time until the symptoms improved was 2 hours after icatibant application in comparison to 11.7 hours after standard therapy (Table 2 (Tab. 2), Figure 7 (Fig. 7)). 

Even the side effects of both therapies were included in the context of the study. The intravenous application of prednisolone/clemastine only rarely led to reactions. Systemic symptoms, however, appeared in single cases of this cohort. One test person complained about symptoms of mild obstructive lung disease, another patient of the same group had increased glucose values, one patient described symptoms of fatigue. In the other group of patients, the local reaction after subcutaneous injection of icatibant played the most important role – 7 patients suffered from pain, in the control group there were only 2 patients after intravenous application. Other reactions at the injection site were reddening, swelling, and thermal sensation; however, systemic reactions did not occur in this group. The study was recently published in the New England Journal of Medicine [94]. The producing company currently applies for approval for this indication. In the USA, another trial is conducted to enlarge the number of patients (NCT01919801). In Germany, icatibant is increasingly applied off-label in patients suffering from acute ACEI-induced angioedema as emergency treatment. According to the results, the treatment of ACEI-induced angioedema is evidence-based. Another result of this study is the proof that the standard therapy (cortisone and antihistamines) is clearly inferior to icatibant and thus also the application in cases of bradykinin-induced angioedema is not indicated. Since an angioedema shows spontaneous self-limitation after about 16 hours, the healing of the edema after 27 hours in the cortisone group is comparable to placebo.

## 6 C1 inhibitor (C1-INH)

Currently 2 human C1 inhibitor concentrates (Berinert^®^ and Cinryze^®^) are available for the treatment of HAE patients. A third C1 inhibitor concentrate (Ruconest^®^) is gained from rabbit milk. The intravenous application of the C1-INH concentrate (Berinert P^®^) is approved for the acute treatment of HAE since many decades and is successfully applied. Off-label it is also used for AAE. Despite the infection risk associated with every blood product, the concentrate turned out to be safe. Cases of viral transmission are not known. The C1-INH concentrate is applied intravenously with a dosage of 20 IE/kg bodyweight. It is applied for acute therapy as well as for prophylaxis. In single cases, also patients with ACEI-induced angioedema were treated with C1-INH concentrates. Recently also a case series of ACEI angioedema was published describing treatment with 1,500 IE Berinert [95]. In the case series, the time of complete healing was 10 hours. In comparison to the treatment with cortisone (33 hours or 27 hours), the treatment with Berinert would be more effective. However, in total only few cases were published internationally [95], [96], [97].

Regarding the patho-mechanism, the effect of C1-INH concentrate in ACEI angioedema is not yet clearly defined. It can be assumed that the treatment with C1-INH reduces further bradykinin production as consequence of an enzyme imbalance. Bradykinin is metabolized by other enzymes such as aminopeptidase 4 and carboxypeptidase. The effect is probably slower than the direct inhibition of the bradykinin receptors by icatibant. A double-blind study for assessment of the efficacy in this indication is performed since 2013 (NCT01843530). Only after finalization and analysis of the results, reliable data may be retrieved. Thus, the treatment of ACEI angioedema with C1-INH is currently not sufficiently evidence-based (Table 3 (Tab. 3)).

## 7 Conclusion

For angiotensin-converting-enzyme inhibitor-induced angioedema, the bradykinin B2 receptor inhibitor icatibant provides a pharmaceutical agent for the first time that is effective according to current studies. However, only few trials have been conducted with this question. The agent is not yet approved officially for this indication and if needed it has to be applied off-label in emergency cases. Corticosteroids and antihistamines do not seem to be effective and are not approved for this indication.

## 8 Allergen-specific immunotherapy (AIT) of respiratory allergies

### 8.1 Pathophysiology and effect mechanism of specific immunotherapy

In the context of AIT, allergen extracts are presented to the immune system as molecule mixture either via the subcutaneous tissue (SCIT = subcutaneous immunotherapy) or via the mucosa (SLIT = sublingual immunotherapy).

Those allergen extracts diffuse first into the local tissue and are absorbed by the local cells and then transported to the local lymph nodes [98], [99]. The bases of AIT are some modulations of the immune system with activation of IgG antibodies that block an allergen antibody-mediated immune response and activation of regulator T cells (Treg) that inhibit a B and T cell mediated immune response to the allergen. Besides, also a cytokine controlled inhibition of the local inflammatory reaction is induced [100], [101], [102], [103], [104].

### 8.2 Specific immunotherapy

The allergen-specific immunotherapy (AIT) is a causal immuno-modulating therapy of respiratory allergies. The application of allergen extracts activates specific blocking antibodies, tolerance inducing cells and messenger substances that inhibit further enhancement of the immune response caused by allergens, that block specific immune responses, and that reduce the inflammatory reactions in the tissue. Clinically, this leads to a reduction of the symptoms and a change of the course of the disease.

Some recent developments have contributed to confirm the position of AIT in the treatment of respiratory allergies. The quality of the allergen extracts is always improved by increasing standardization [105]. Basic research led to further clarification of the pathophysiology of respiratory allergies and the mode of action of specific immunotherapy [106], [107]. The implementation and official approval of the sublingual immunotherapy (SLIT) beside subcutaneous immunotherapy (SCIT) also belongs to the important milestones of AIT [108]. A recently published S2k AWMF practical guideline summarizes the study situation on AIT and the efficacy of different agents [109].

Economically, allergic rhinitis and its subsequent diseases (such as bronchial asthma) cause enormous direct and indirect costs. Accordingly, therapeutic options, especially AIT, are assessed socio-economically based on cost-benefit-effectiveness analyses [110], [111].

In long-term evaluations, AIT is significantly more cost-effective in comparison to symptomatic pharmacotherapy in allergic rhinitis and allergic bronchial asthma [112].

Meta-analyses clearly confirm the effectiveness of SCIT and SLIT for certain allergens and age groups. Data of controlled studies differ in view of their quantity, their quality, and dosage schemes and require drug-specific assessment [113].

Allergen extracts for SCIT or SLIT cannot be compared because of their heterogenic composition and different measuring methods of the effective ingredients. For SCIT, non-modified allergens are applied as aqueous or physically coupled (semi-depot) extracts as well as chemically modified extracts (allergoids) as semi-depot extracts. The allergen extracts for SLIT are administered as aqueous solutions or pills [114].

It is recommended to assess the single pharmaceutics according to clearly defined criteria. On the website of the German Society of Allergology and Clinical Immunology (DGAKI, Deutsche Gesellschaft für Allergologie und Klinische Immunologie), tables can be found showing a drug-specific description of the AIT products available in Germany, Switzerland, and Austria (http://www.dgaki.de/leitlinien/s2k-Leitlinie-sit/) (Table 4 (Tab. 4), Table 5 (Tab. 5), Table 6 (Tab. 6), Table 7 (Tab. 7)).

The tables list the trials and their assessment criteria for adults and children. The efficacy of SCIT in the context of pollen-associated allergic rhino-conjunctivitis in adults is very well proven by numerous studies. The efficacy of drugs for dust mite allergy is well proven by some studies. However, the trial situation for children regarding both types of allergies is comparably poor.

The efficacy of SLIT in the treatment of allergic rhino-conjunctivitis caused by grass pollen in adults and children is very well confirmed and for tree pollen allergy it is well proven in adults. For dust mite allergy, new controlled trials with sometimes high patient populations confirm the efficacy of SLIT in adults. For patients with allergic rhino-conjunctivitis, SCIT or SLIT can be performed with pollen or mite allergen extracts. The efficacy of this therapy was confirmed by at least one double-blind placebo-controlled trial [114].

The efficacy of AIT has been confirmed by numerous meta-analysis with many trials [114]. Calderon et al. evaluated 33 clinical studies on AIT in patients suffering from grass pollen allergy who met the predefined criteria. Dretzke et al. published a meta-analysis in 2013 evaluating 28 trials on allergic rhinitis [114].

Regarding dust mite allergy, a systematic review article on the efficacy and tolerance of SCIT and SLIT was published in 2013 encompassing 44 trials [115].

## 9 Conclusion

In summary, those meta-analysis and review articles confirm a well-documented efficacy of AIT. Because of the heterogeneity of the studies and the difficulty of direct comparison, a general recommendation cannot be given. The efficacy of each pharmaceutical product must be confirmed by specific trials.

## 10 Outlook

On the one hand, the presented cases show that there are still diseases in ENT that are not treated in an evidence-based way because of missing studies. On the other hand, those cases also show how difficult it is to plan, finance, and finally conduct acknowledged high-quality trials.

For planning and conducting such studies the cooperation with several university hospitals is required. Beside the motivation of single colleagues also the infrastructure and professional study centers must be provided. The new study center of the German ENT Society could be such a platform. I would like to encourage motivated colleagues to perform research especially in those fields where evidence gaps are obvious.

## Notes

### Competing interests

The author declares to have received research support by the companies Shire (Firazyr^®^) and Behring (Berinert^®^).

## Figures and Tables

**Table 1:**
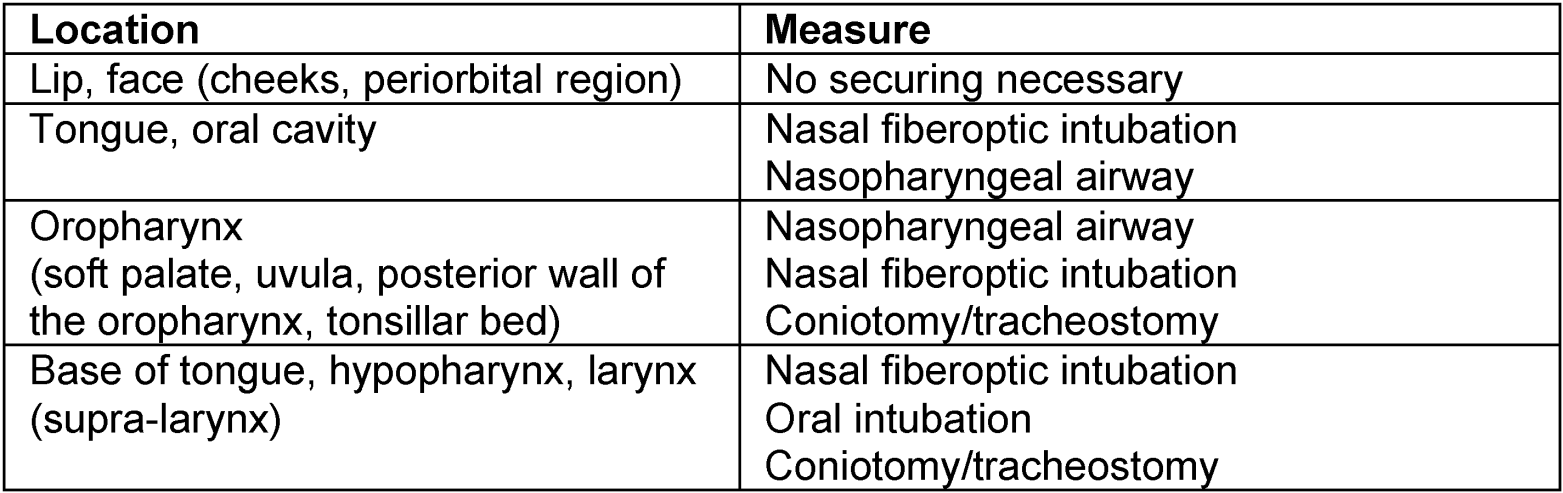
Applied methods for mechanical protection

**Table 2:**
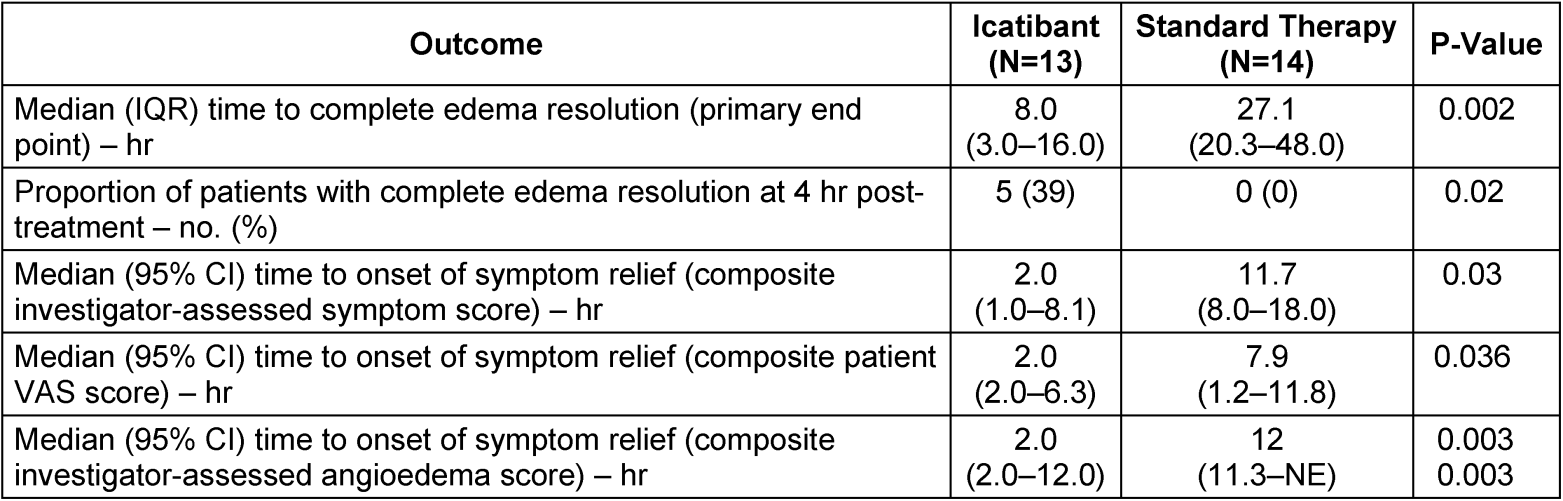
Results of the AMACE trial

**Table 3:**
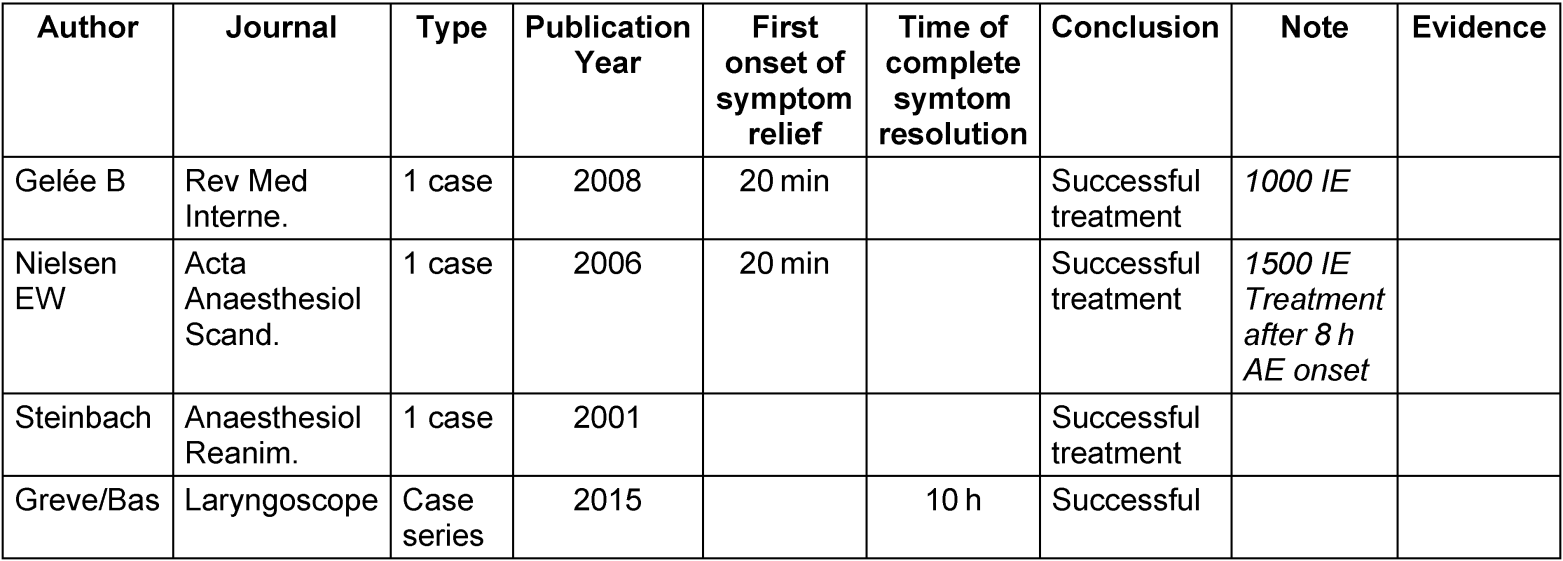
Published casuistics and case series on the application of Berinert in ACEI-induced angioedema

**Table 4:**
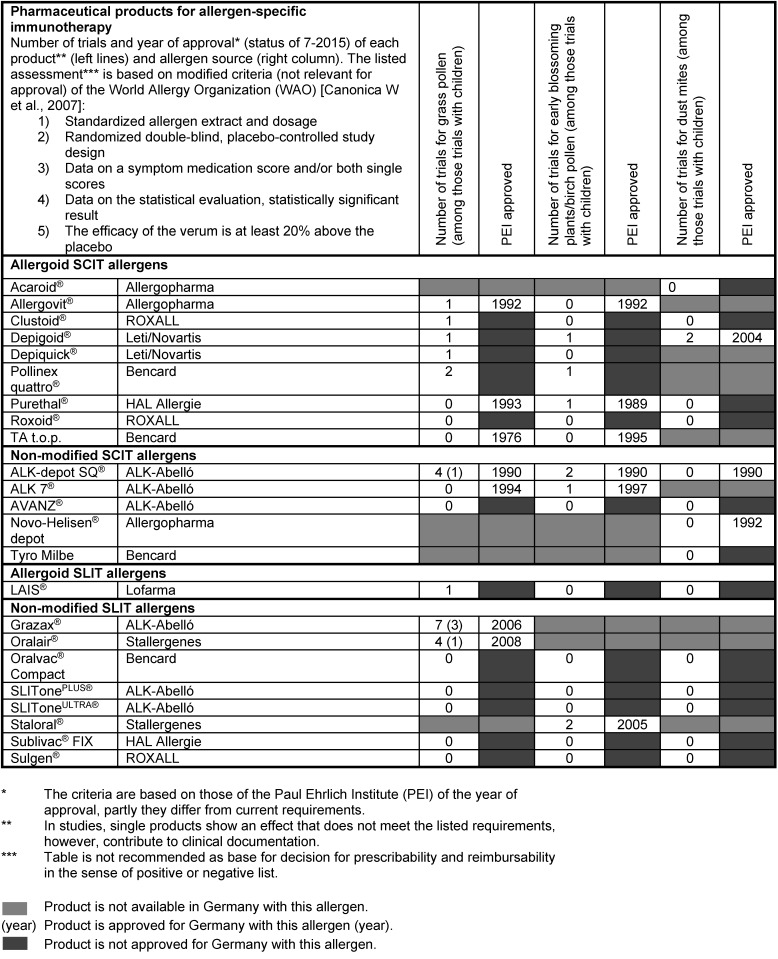
Pharmaceutical products for AIT with the number of the specific studies and the status of approval (source: website of DGAKI, www.dgaki.de/Leitlinien/s2k-Leitlinie-sit/)

**Table 5:**
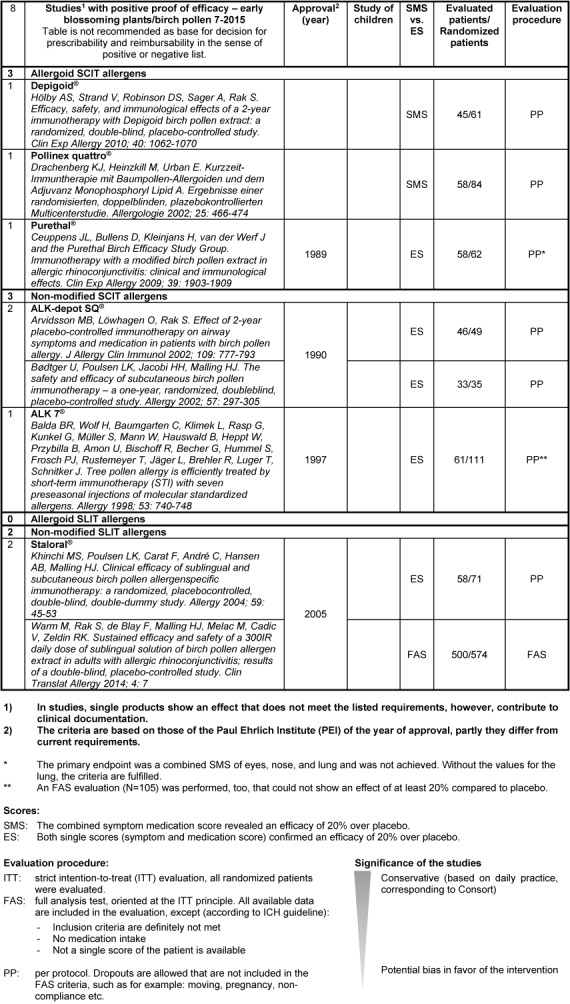
Studies with positive proof of efficacy for early blossoming plants (birch pollen) (source: website of DGAKI, www.dgaki.de/Leitlinien/s2k-Leitlinie-sit/)

**Table 6:**
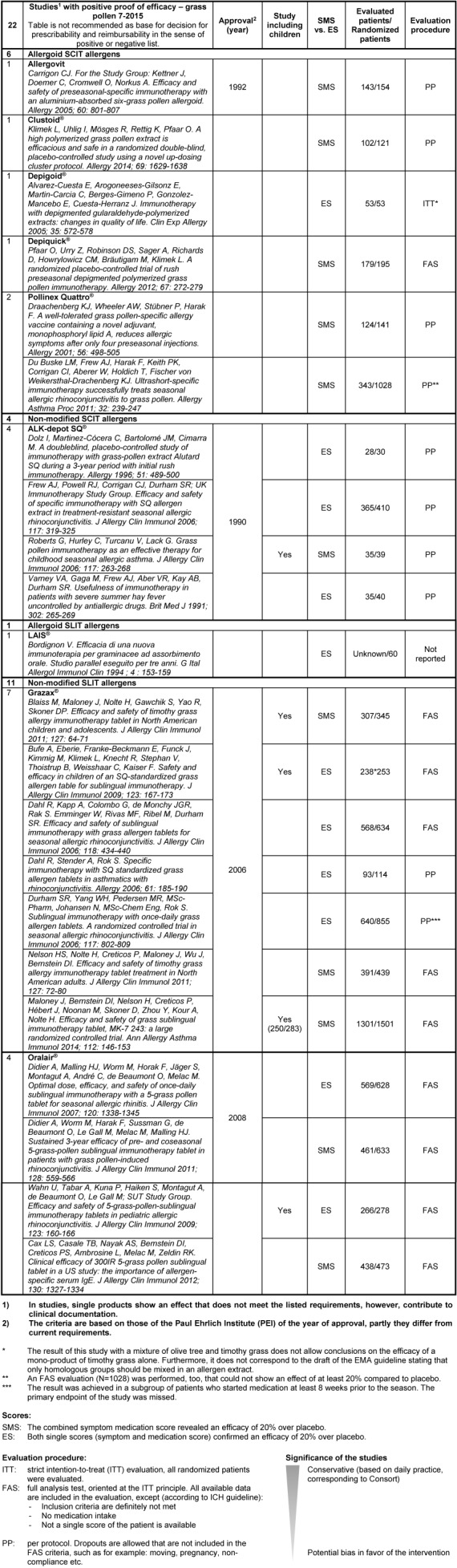
Studies with positive proof of efficacy for grass allergies (source: website of DGAKI, www.dgaki.de/Leitlinien/s2k-Leitlinie-sit/)

**Table 7:**
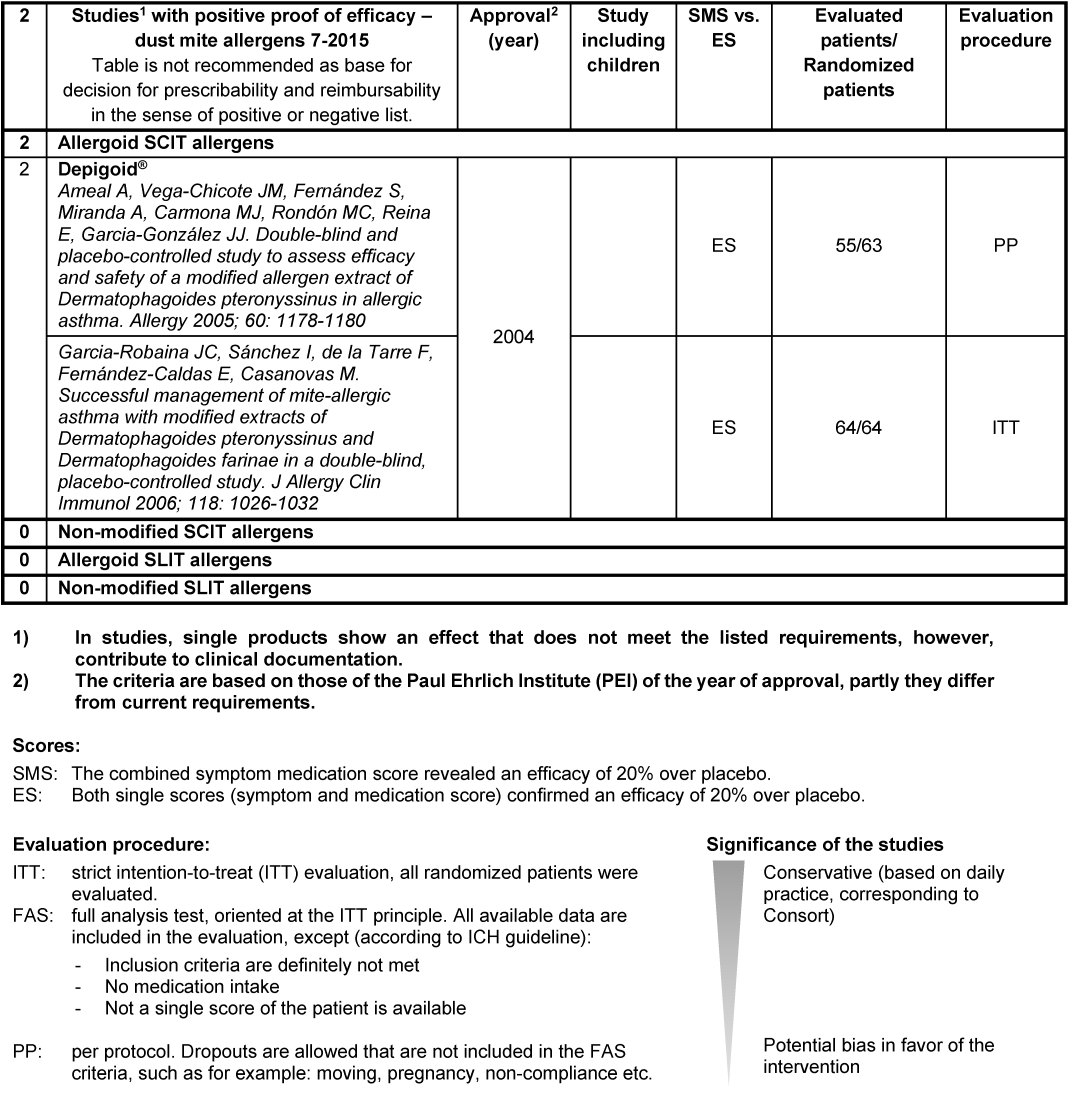
Studies with positive proof of efficacy for dust mite allergies (source: website of DGAKI, www.dgaki.de/Leitlinien/s2k-Leitlinie-sit/)
